# Toxicity of metal-based nanoparticles: Challenges in the nano era

**DOI:** 10.3389/fbioe.2022.1001572

**Published:** 2022-11-10

**Authors:** Naiding Zhang, Guiya Xiong, Zhenjie Liu

**Affiliations:** ^1^ Department of Vascular Surgery, 2nd Affiliated Hospital, School of Medicine, Zhejiang University, Hangzhou, Zhejiang, China; ^2^ Department of Science and Research, 2nd Affiliated Hospital, School of Medicine, Zhejiang University, Hangzhou, Zhejiang, China

**Keywords:** metal-based nanoparticle, physicochemical property, nanotoxicity, mechanism, assessment, mitigation, regulatory movement

## Abstract

With the rapid progress of nanotechnology, various nanoparticles (NPs) have been applicated in our daily life. In the field of nanotechnology, metal-based NPs are an important component of engineered NPs, including metal and metal oxide NPs, with a variety of biomedical applications. However, the unique physicochemical properties of metal-based NPs confer not only promising biological effects but also pose unexpected toxic threats to human body at the same time. For safer application of metal-based NPs in humans, we should have a comprehensive understanding of NP toxicity. In this review, we summarize our current knowledge about metal-based NPs, including the physicochemical properties affecting their toxicity, mechanisms of their toxicity, their toxicological assessment, the potential strategies to mitigate their toxicity and current status of regulatory movement on their toxicity. Hopefully, in the near future, through the convergence of related disciplines, the development of nanotoxicity research will be significantly promoted, thereby making the application of metal-based NPs in humans much safer.

## Introduction

Nanoparticles (NPs) are nanoscopic particles ranging from 1 to 100 nm, comprising materials such as carbon, metals, metal oxides, polymers and so on (Lee and Jun 2019; Ferreira Soares et al., 2020; Diez-Pascual, 2021; Qi et al., 2021). Compared to the corresponding bulk, nanoscale materials significantly change their physicochemical, mechanical, and biological properties (Chen et al., 2016; Saifi et al., 2018). The NPs impart many advantages like improved bioavailability and prolonged residence time depending on the small size and surface functionalities (Gwinn and Vallyathan, 2006; Mudshinge et al., 2011). These unique material characteristics make NPs a hotspot of materials science research and commercial/industrial interest (Ragelle et al., 2017; Kumari et al., 2020).

Nanotechnology is a revolutionary science involving fabricating and dealing with nanometer particles of various materials. Nanotechnology has endowed us with a new robust platform with a wide range of potential and practical applications including medicine, diagnostic devices, agriculture, catalysts, cosmetics, biological sensors, and so on (Missaoui et al., 2018; Bayda et al., 2019; He et al., 2019; Puglia and Santonocito, 2019; Zhou et al., 2021). As an important component of engineered NPs, metal-based NPs are generally made of metal precursors, including metal NPs and metal oxide NPs (Makhdoumi et al., 2020). Nowadays, metal-based NPs have received increasing attention due to their different properties such as large surface area, optically active, mechanically strong and chemically reactive (Khan et al., 2019). Owing to these properties, metal-based NPs have been widely used in biomedical application. It holds the promise to treat difficult and poor prognostic diseases such as cancers, complex infections, autoimmune diseases, and other clinical problems (Shen et al., 2018; Singh et al., 2018; Kalashnikova et al., 2020; Rashki et al., 2021). Compared to conventional therapeutics, nanoparticle-based therapy has more advantages in terms of cell specificity and selectivity, targeted delivery, high transport efficiency, and improved biopharmaceutical properties (Ahmad et al., 2018). NPs can significantly improve drug efficacy by altering the rates of drug metabolism and clearance (Seleci et al., 2017; Ravindran et al., 2018). Moreover, conjugated with targeting moieties such as specific antibodies and ligands, metal-based NPs can be actively targeted to the disease sites by recognizing and binding to the corresponding membrane proteins (Meka et al., 2019; Zhang et al., 2020). Thus, specific metal-based nanoparticle-based therapeutics have already been approved by the United States Food and Drug Administration (FDA) (Bobo et al., 2016).

While the increasing need for novel drugs or drug-release systems has led to vast advancements in nanomedicine, some safety concerns exist (Liu et al., 2020a; Najahi-Missaoui et al., 2020). While more and more metal-based NPs have been researched in various applications, information on the impact of NPs on human body is lagging behind (Najahi-Missaoui et al., 2020). It is suggested that NPs have a greater risk of toxicity than the corresponding bulk (Hoet et al., 2004). After metal-based NPs exposure, several side effects such as immunogenic reactions, nephrotoxicity, and neurotoxicity have been observed (Makhdoumi et al., 2020). It is also well known that high reactivity, longer circulation time, off-target nanomaterial accumulation, and unintentional exposure are the main risks of NP toxicity (Johnston et al., 2010; Yokel et al., 2012; Saifi et al., 2018). Although numerous toxicological studies have been conducted on metal-based NPs, the potential acute and chronic hazards to humans are not yet fully elucidated. It is necessary to thoroughly understand the basis of metal-based NP toxicity before extensive metal-based NPs can be safely applied to human beings.

This review briefly introduces the physicochemical properties of metal-based NPs that affect toxicity. Then the discussion of the mechanisms of metal-based NP toxicity will provide an overview of how metal-based NPs interact with the body. This review also covers well-established methodologies for nanoparticle toxicological assessment and evaluation *in vitro* and *in vivo*. Furthermore, we summarize some potential strategies to mitigate metal-based NP toxicity for safer biomedical applications in humans. The last section of the review presents current status of regulatory movement on nanotoxicity. We hope this review could improve our understanding of metal-based NP toxicity and guide the development of strategies to mitigate potential hazards to humans.

## Physicochemical properties of metal-based nanoparticles that influence toxicity

**FIGURE 1:**
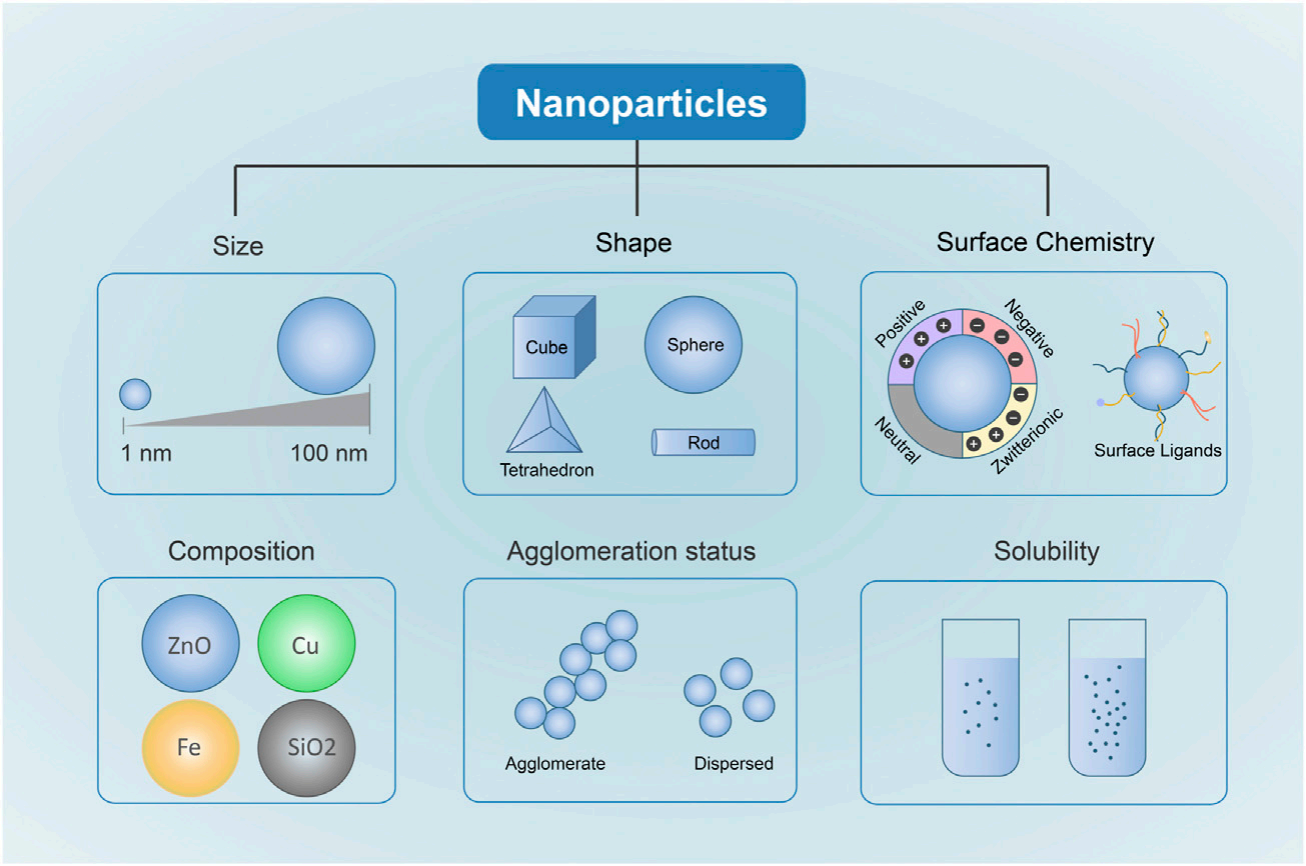
Physicochemical Properties Affecting metal-based NP Toxicity. Different Sizes, shapes, chemical compositions, surface chemistry, solubility and agglomeration are closely related to the toxicity induced by metal-based NPs.

The physical and chemical properties of NPs act as key factors affecting NP uptake, translocation and accumulation in live tissues, which determines the fate of NPs and their mechanisms of toxicity (Zoroddu et al., 2014). In addition, some studies have confirmed that the physicochemical properties of NPs can dramatically affect nano-bio interactions and the potential toxicity (Kreyling et al., 2009; Attarilar et al., 2020; Manuja et al., 2021). Understanding these physicochemical properties has important implications for designing and fabricating safer metal-based NPs. The toxicity of metal-based NPs depends on their physicochemical properties, such as chemical composition, size, shape, and surface chemistry (Figure 1) (Wu and Tang, 2018).

Particle size is a crucial physicochemical property contributing to cytotoxicity (Saifi et al., 2018; Donahue et al., 2019). NP size plays an important role in determining the approach of internalization in the cells, which finally influences the NP distributions in live tissues and mechanisms of nano-bio interactions (Sayes et al., 2007; Sukhanova et al., 2018). Similar to the nanosize of protein globules, DNA helix and cell membrane thickness, NPs can easily penetrate through the cell membrane and enter cells and cell organelles (Sukhanova et al., 2018). Furthermore, it is demonstrated that NPs with smaller size can pass through cell membranes by translocation, whereas NPs with larger size enter cells by other transportation mechanisms like phagocytosis, micropinocytosis, and non-specific translocation (Zhang et al., 2015). The smaller size of NPs enables higher permeability of cell membranes to interact with organelles such as mitochondria, lysosomes, and nucleus, finally causing cell damage (Donahue et al., 2019). In MCF-7 breast cancer cells, gold (Au) NPs smaller than 10 nm (2 and 6 nm) had the deeper internalization into the cell nucleus, while larger NPs (10 and 16 nm) were only accumulated in the cytoplasm (Huo et al., 2014). Some studies also revealed that the size of Titanium dioxide (TiO_2_) NPs correlated with their cytotoxicity (Kim et al., 2014; Wang et al., 2019). It was reported that smaller TiO_2_ NPs (6 nm) exposure under illumination caused more oxidative stress and more DNA damage than larger NPs (12 and 15 nm) in developing zebrafish embryos (Kim et al., 2014). More importantly, a kinetic study showed that organ distribution of NPs is highly size-dependent (De Jong et al., 2008). The 10 nm Au NPs had the most extensive organ distribution including blood, liver, spleen, kidney, testis, thymus, heart, lung, and brain, while the larger NPs were only found in blood, liver, and spleen. Silver (Ag) NPs with a size of 10 nm had higher tissue distribution and caused more severe hepatobiliary toxicity than larger NPs (40 and 100 nm) (Recordati et al., 2016). Moreover, the NP size could also have an impact on the mechanism of NP toxicity. After Au NPs exposure in four cell lines, the cellular response depends on the NP size, for the reason that 1.4 nm NPs cause cell death by necrosis whereas 1.2 nm NPs cause programmed cell death by apoptosis (Pan et al., 2007). Additionally, the smaller size of NPs leads to a larger surface area to volume ratio, which could increase the reactivity of NPs as it has more surface area to interact with cellular components (Johnston et al., 2010).

Along with NP size, NP shape can significantly affect NP toxicity (Sukhanova et al., 2018; Demir, 2021). NPs have various shapes including spheres, ellipsoids, cylinders, sheets, cubes, and rods (Sukhanova et al., 2018). For NPs of similar size and composition, their shape of NPs can alter their biological activities, including biodistribution, cellular uptake, deposition, and clearance (Zare et al., 2021). It is evident that the NP shape modulates the endocytosis kinetics of NPs in the efficient coarse-grained molecular dynamics (CGMD) model (Huang et al., 2013). By local energy analyses, the NP shape could affect the symmetry of curvature energy and hence determine the endocytic pathways and the angle of entry during the endocytosis process. Furthermore, there is evidence that non-spherical NPs are internalized by cells at faster rates and in larger amounts than spherical NPs (Hadji and Bouchemal, 2022). Numerous studies have shown that non-spherical NPs had longer circulation time in the blood and higher accumulation in specific organs than spherical ones (Geng et al., 2007; ArnidaJanat-Amsbury et al., 2011; Chu et al., 2013). For instance, rod-shaped PEGylated Au NPs had longer circulation time and higher accumulation in the tumors, compared to spherical NPs (ArnidaJanat-Amsbury et al., 2011). Similarly, the accumulation of discoidal porous silicon nanovectors into the tumor mass of breast cancer bearing mice was 5 times higher than spherical ones with similar size (Godin et al., 2012). Moreover, changes in shape may also significantly alter the surface area, which plays a role in determining their toxicity. Compared with sphere-shaped Iron (III) oxide (Fe_2_O_3_) NPs, rod-shaped Fe_2_O_3_ NPs were found to cause more severe toxic effects in mouse macrophage cells (RAW 264.7) with higher levels of lactate dehydrogenase (LDH) and tumor necrosis factor-α (TNF-α), reactive oxygen species (ROS) generation and necrosis (Lee et al., 2014). In addition, rod-shaped Cerium oxides (CeO_2_) NPs produced higher toxic responses with higher extracellular LDH release and pro-inflammatory cytokine TNF-α production in the RAW264.7 cell line, compared to cubic/octahedral-shaped NPs with similar chemical composition and crystallinity (Forest et al., 2017). On the contrary, the results of toxicity comparison of three kinds of CeO2 NPs with different shapes (cube-, octahedron-, and rod-like) in human hepatocellular carcinoma cells reported that cube-like NPs caused highest cytotoxicity and rod-like NPs caused lowest cytotoxicity (Wang et al., 2015). Furthermore, spherical TiO_2_ NPs exhibited 5-fold increased mortality in the *Escherichia coli* than elongated TiO_2_ (Simon-Deckers et al., 2009). It may be that different shapes of metal-based NPs lead to different bioaccumulation or/and reactivity, finally resulting in the differences in toxicity (Dai et al., 2015).

Besides size and shape, surface chemistry is another critical factor that remarkably affects toxicity (Chandran et al., 2017). The surface charge is closely related to NP toxicity, which can affect NP pharmacokinetics and their interactions with organelles and biomolecules. Zeta potential (ζ-potential) has already been adopted to characterize the surface charge of NPs and to predict NP toxicity. Numerous studies show that NPs with positive zeta potential are more prone to impart toxicity than those with negative zeta potential (Li et al., 2013; Shahbazi et al., 2013; Roshanzadeh et al., 2020; Sinclair et al., 2021), partially because that NPs have higher electrostatic interactions to negatively charged cellular membranes, thus increasing cellular uptake of NPs, leading to more cell damages (Albanese et al., 2012; Frohlich, 2012; Chusuei et al., 2013; Jeon et al., 2018; Roshanzadeh et al., 2020). For instance, comparison of the cytotoxicity of positively and negatively charged Au NPs revealed that the positively charged NPs were more toxic, due to their enhanced uptake (Huhn et al., 2013). Furthermore, magnetic NPs of Ferroferric Oxide (Fe_3_O_4_), oleic acid-coated Fe_3_O_4_, and carbon-coated Fe with different surface charges exhibited different cytotoxic effects on human hepatoma BEL-7402 cells and increased NP surface charges caused higher cytotoxicity by cell cycle arrest and inducing apoptosis (Kai et al., 2011). The phenomenon can be explained as the higher positive charge of NPs results in more significant electrostatic interactions with cells. The longer and greater the electrostatic interactions between NPs and cells, the more endocytic uptake of NPs into cells (Chusuei et al., 2013). In the systemic circulation, metal-based NPs could absorb a variety of proteins like albumin, immunoglobin, and other functional biomolecules to the surface of metal-based NPs to form a “protein corona”, which has been reported to alter the surface properties of metal-based NPs (Duran et al., 2015; Choi et al., 2017; Barbalinardo et al., 2018). On the one hand, NP-protein corona formation can change critical physicochemical characteristics of NP surface, thereby influencing pharmacological and toxicological characteristics of NPs (Ahsan et al., 2018; Ovais et al., 2020; Mishra et al., 2021). On the other hand, the configuration of those proteins absorbed on the NP surface would be altered, which leads to a change in their functional activity and biological processes (Tomak et al., 2021). Recent studies demonstrated that NP-protein corona formation could interact with many immune system components to either stimulate or suppress the immune response and cytotoxic effects (Hu et al., 2011a; Ruge et al., 2011; Lee et al., 2015; Lu et al., 2018). In addition, various coating materials like polyethylene glycol (PEG), poly (lactic-co-glycolic acid) (PLGA), polylactic acid (PLA), lipids, and others have been used to modify the metal-based NP surface, which considerably changes the physicochemical properties of NPs (Tefft et al., 2015; Oertel et al., 2016; Guerrero et al., 2019; Jiao et al., 2020; Murphy et al., 2021). Surface modifications of NPs not only affect their pharmacokinetics and change their biodistribution, clearance, and elimination, which are closely related to their toxicity in the body (Arami et al., 2015; Das et al., 2017; Yu et al., 2018a). For NPs with similar chemical compositions, different surface modifications lead to toxic effects in varying degrees (Zhang et al., 2021). Among three types of Aluminium Oxide (Al_2_O_3_) NPs including pristine Al_2_O_3_ NPs (p- Al_2_O_3_), hydrophilic (w- Al_2_O_3_), and lipophilic (o- Al_2_O_3_), it was found that o-Al_2_O_3_ NPs were more toxic than p-Al_2_O_3_ and w-Al_2_O_3_ NPs both *in vitro* and *in vivo*, as a result of cell membrane damage and over-production of ROS. Moreover, some studies illustrated that some specific modifications of NP surface could mitigate the toxicity of NPs and make them tissue-specific (Das et al., 2017; Lai et al., 2021). For example, after coated with a silica layer, the zinc dioxide (ZnO) NPs exhibited less cytotoxicity in human dermal fibroblast cells with less enzyme leakage, ROS production, and oxidative stress than their bare NPs, as a result of the surface modification restricting formation of free radicals and the release rate of zinc ions, as well as reducing the surface interactions of ZnO NPs with cells (Ramasamy et al., 2014).

The chemical composition and crystal structure of NPs are also critical to determine their toxicity. NPs fabricated by different materials differ in the mechanisms of toxicity. In one comparative study of the toxicity of carbon black, single-wall carbon nanotube, silicon dioxide (SiO_2_), and ZnO NPs, ZnO induced the most remarkable cytotoxicity by promoting intracellular oxidative stress, whereas SiO_2_ NPs mainly induced DNA damage (Yang et al., 2009). This result revealed that chemical composition is an important factor in the toxic effects of different NPs. Besides, toxicity screening performed on several types of metal oxide NPs observed that CuO and ZnO NPs induced the highest toxicity in acute models both *in vitro* and *in vivo*, mainly due to significant promotion of pro-inflammatory cytokines release and inhibition of macrophage viability (Areecheewakul et al., 2020). It is indicated that the chemical composition of metal-based NPs may influence the severity and mechanism of NP toxicity. Additionally, crystal structure can also affect metal-based NP toxicity (Vandebriel et al., 2018). Despite of same chemical composition, NPs with different crystal structures have different toxic responses. Compared to the anatase structure of TiO_2_ NPs, TiO_2_ NPs with rutile structure were more cytotoxic in cultured BEAS-2B cells by inducing hydrogen peroxide and oxidative DNA damage (Gurr et al., 2005). However, the NP crystal structure may be changed in different environments (Zhang et al., 2003). During the coalescence process, the crystal structure of Ag NPs was observed to transform from the common fcc structure to the unusual hcp structure (Grouchko et al., 2009).

Apart from those factors mentioned above, the solubility and agglomeration also affect the NP toxicity. Various intrinsic and extrinsic factors play a role in the solubility of the NPs. The intrinsic physicochemical properties like size, shape, surface chemistry, and crystal structure can affect NP dissolution process (Borm et al., 2006; Zhang et al., 2011; Misra et al., 2012a). Also, the extrinsic factors of surrounding media such as PH, ionic strength and water hardness can influence the NP behavior in solution (Peretyazhko et al., 2014; Fernando and Zhou, 2019). Increasing number of studies illustrate that the solubility of the NPs in surrounding media significantly influences their bioavailability, internalization process and toxicity mechanisms (Misra et al., 2012b). Furthermore, it has been suggested the metal ions release of metal-based NPs in media mainly contribute to the NP toxicity (Wang et al., 2016). For instance, a toxicological evaluation of ZnO NPs in various media showed that the NP toxicity in five types of media deceased as follows: ultrapure water >0.85% NaCl > minimal Davis > Luria-Bertani > phosphate-buffered saline (Li et al., 2011). The reason for the toxicity order is attributed to the decrease of the concentration of Zn^2+^ ions by the generation of precipitates and zinc complexes in different media. There is evidence that the degree of NP agglomeration also has an effect on the NP exposure, uptake, and distribution in the living tissues, thus influencing the toxicity of NPs (Creutzenberg et al., 2012; Bruinink et al., 2015). Agglomeration status refers as a collection of a group of NPs *via* weak forces, like van der Waals forces or electrostatic forces. Besides, various factors influencing agglomeration process in solution mainly include size, shape, surface structure, chemical composition, and so on (Ahamed et al., 2008; Bae et al., 2010; Andersson et al., 2011). Moreover, agglomeration status highly depends on the environmental parameters, like temperature, PH, and solution chemistry (Guzman et al., 2006; Karakoti et al., 2008; Bruinink et al., 2015). For example, in an *in vitro* testing, large agglomerates of 17 nm TiO_2_ caused stronger toxicity responses including glutathione depletion, IL-8 and IL-1β increase, and DNA damage in monocytic cell lines, compared to small agglomerates. However, it was observed that large agglomerates of 117 nm TiO_2_ caused higher pulmonary cytotoxicity in the mice inhalation study and more blood DNA damage in gavaged mice than small agglomerates (Murugadoss et al., 2020). Interestingly, large agglomerates do not exhibit less toxicity than small agglomerates.

## Mechanisms of metal-based nanoparticle toxicity

**FIGURE 2:**
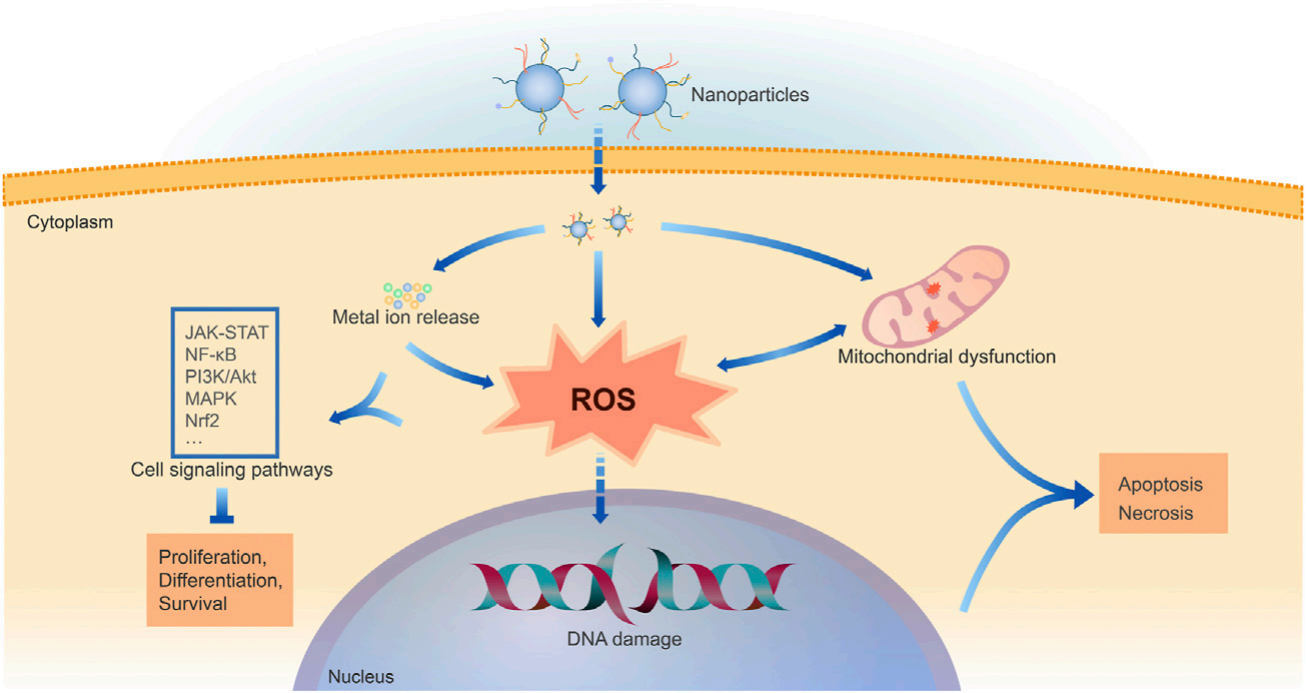
Mechanisms of cell damage by metal-based NPs. Following cellular uptake of NPs, metal-based NPs can release intracellular metal ions and induce oxidative stress directly or through mitochondrial dysfunction. ROS generation and consequent oxidative stress are shown to be the fundamental cause of metal-based NP toxicity. The excessive ROS generation and metal ions impact multiple cell signaling pathways, mainly including JAK-STAT, NF-κB, PI3K/Akt, MAPK, Nrf2. ROS can also cause DNA damage. Thus, it hinders cell physiological processes like proliferation and differentiation, eventually leading to cell apoptosis or necrosis.

The physicochemical properties determine the metal-based NP toxicity. Various *in vitro* and *in vivo* models have already been adopted to assess the toxicity of NPs, including cell lines, 3D cell culture, zebrafish, rodents, and others (Bahamonde et al., 2018; De Simone et al., 2018; Haque and Ward, 2018; Saifi et al., 2018; Xu et al., 2019; Cao et al., 2021). The exact mechanism of NP toxicity remains unclear. Multiple mechanisms contribute to the metal-based NP toxicity, including oxidative stress *via* ROS generation, mitochondrial dysfunction, DNA damage, and others (Figure 2) (Khanna et al., 2015; Nemmar et al., 2016; Sukhanova et al., 2018).

The recent studies correlate the NP toxicity with the consequences of ROS generation (Wang et al., 2017; Angele-Martinez et al., 2022). Most work has shown that ROS generation and consequent oxidative stress are the fundamental cause of NP toxicity (Abdal Dayem et al., 2017; Makhdoumi et al., 2020; Mishra and Panda, 2021). Various metal-based NPs have already been discovered to induce toxicity by generating ROS in the living body (Yu et al., 2018b; Horie and Tabei, 2021). In cells of the normal state, there is a balance between the generation and removal of ROS. ROS mainly includes hydrogen peroxide, hydroxyl radical, singlet oxygen, hypochlorous acids, and superoxide anion, which are crucial triggers of oxidative stress (Poyton et al., 2009; Jakubczyk et al., 2020; Mishra and Panda, 2021). Under physiological conditions, ROS plays a vital role in regulating various aspects of cell behaviors, including cell proliferation, differentiation, and death (Forrester et al., 2018; Jakubczyk et al., 2020). However, the disruption of redox balance may result from increased ROS generation and/or decreased levels of antioxidants thus elevated ROS level results in oxidation of macromolecules, such as proteins, lipids, and nucleic acids, which subsequently leads to DNA damage, disturbed signal transduction, cytotoxicity, and cell death (Ray et al., 2012; Mishra and Panda, 2021).

NP-induced oxidative stress can mainly be divided into two types according to their underlying mechanisms (Horie and Tabei, 2021). One type is direct oxidative stress, also called primary oxidative stress. It is illustrated that the higher surface area of NPs is associated with more reactive sites on the surface, resulting in higher chemical reactivity to increase ROS generation (Wilson et al., 2002; Fu et al., 2014). The reactive surface with oxidants and free radicals can also significantly accelerate the formation of ROS. For instance, surface-bound free radicals on crystalline silica particles can enhance oxidative stress *via* a surface reaction to generate ROS (Fubini and Hubbard, 2003). Besides, studies have shown that some particular metal-based NPs, such as TiO_2_ NPs can generate ROS through a photocatalytic process under light irradiation (Toloman et al., 2019; Balaji et al., 2020; Romolini et al., 2021). Additionally, transition metals in NPs such as copper (Cu), and chromium (Cr) can participate in ROS generation *via* Fenton and Haber-Weiss reactions, ultimately enhancing oxidative stress (Huang et al., 2010; Angele-Martinez et al., 2017). Another type is indirect oxidative stress, also called secondary oxidative stress. In this type, NPs are not the direct cause of oxidative stress. Mitochondria are crucial organelles involved in NP-induced oxidative stress. Intracellular metal ions released from internalized metal-based NPs can depolarize the mitochondrial membrane and disturbance of electron-transport chain, eventually leading to mitochondrial dysfunction (Fu et al., 2014; Hosseini et al., 2014). In mitochondria, molecular oxygen is consumed to synthesize adenosine triphosphate *via* a series of coupled proton and electron transfer reactions in the physiological process. However, as a result of metal-based NP exposure, the mitochondrial electron-transport chain (METC) would be disrupted, thus resulting in the elevation of intracellular ROS level (Ashrafi Hafez et al., 2019). For cancer-specific cytotoxicity, superparamagnetic iron oxide NPs could specifically target the METC complexes, resulting in mitochondrial ROS production, which induced cancer cell death (He et al., 2016). NP-induced ROS generation could also be derived from the interaction of metal oxide NPs with ROS-associated enzymes and receptors. For instance, in the presence of TiO_2_ NPs, NADPH oxidase was hyper-activated, and the subsequent increase in ROS generation induced a higher level of oxidative stress (Masoud et al., 2015). In addition, ROS generation by phagocytes, like macrophages and neutrophils, is also the cause of NP-induced oxidative stress (Horie and Tabei, 2021). In the zebrafish model, exposure to Ag NPs would damage the mitochondria of innate immune cells including macrophages and neutrophils, leading to excessive ROS production, which finally induced innate immune toxicity (Chen et al., 2021). Notably, a recent study demonstrated that co-exposure to gold (Au) NPs and lipopolysaccharide would result in upregulating extracellular ROS generation by hepatic macrophage, theraby inducing hepatic apoptosis and aggravating liver injury in mice (Yang et al., 2022). After internalized by macrophages, Fe_2_O_3_ NPs can be enclosed and hence biodegraded in the lysosomes, and released atomic iron participates in ROS production *via* Fenton reaction. More importantly, ROS appears to involve in the Fe_2_O_3_ NP-induced macrophage activation through several signal pathways, consequently regulating the innate immune response (Mulens-Arias et al., 2021). Furthermore, the higher level of intracellular ROS may damage the mitochondrial membrane and the electron-transport chain, leading to the more ROS and amplifying the oxidative imbalance, also referred to as ROS-induced ROS release (Zorov et al., 2014; Mishra and Panda, 2021). However, not all NP-induced toxicity is mediated *via* ROS. A comparison study focused on cytotoxicity, DNA damage, and oxidative stress of different metal oxide NPs (CuO, TiO_2_, ZnO, CuZnFe_2_O_4_, Fe_3_O_4_, Fe_2_O_3_) in human lung epithelial cell line A549. Among these metal-based NPs, only CuO NPs caused oxidative stress by significantly increasing intracellular ROS (Karlsson et al., 2008).

It has been reported that the excessive ROS generation and metal ions released from internalized metal-based NPs have an impact on multiple cell signaling pathways, mainly including Janus kinase/signal transducers and activators of transcription (JAK-STAT), nuclear factor-kappa-lightchain enhancer of activated B cells (NF-κB), Phosphatidylinositol 3-kinase (PI3K)/protein serine threonidase (Akt), mitogen-activated protein kinase (MAPK), and nuclear factor erythroid 2-related factor 2 (Nrf2) (Ko et al., 2016; Chang et al., 2017; Kim et al., 2017; Mahmoud et al., 2019; Chang et al., 2021). Activation or inhibition of these signaling pathways can impact many significant physiological processes like cell proliferation, differentiation, survival, and immune regulation (Li and Tang, 2020). In HeLa cells, the Ag-NP-hydrogel exposure activated JAK-STAT signaling pathway by upregulating JAK-STAT cascade-related gene, thereby inducing toxicity (Xu et al., 2012). Silica NPs could induce oxidative stress, inflammation, and NO/NOS system disorder, ultimately causing endothelial cytotoxicity by activating the MAPK/Nrf2 and NF-κB signaling pathways (Guo et al., 2015). After intratracheal instillation in male Wistar rats, nickel oxide (Nio) NPs activated the NF-κB pathway by upregulation of mRNA and protein expression of NF-κB, an inhibitor of κB kinase-α and nuclear factor-inducing kinase, partially causing pulmonary damage (Chang et al., 2017). It was demonstrated that SiO_2_ NPs induced the lung alveolar epithelial cell apoptosis by ROS-regulated PI3K/AKT-mediated mitochondria- and ER stress-dependent signaling pathways, leading to lung injury (Lee et al., 2020). However, no single mechanism or signaling pathway has been yet found to elucidate the NP toxicity fully. Furthermore, the crosstalk between different signaling pathways complicates the exact mechanism of NP toxicity.

## Assessment of metal-based nanoparticle toxicity

With the rapid development and wide application of nanotechnology and nanomaterials, the impact of metal-based NPs on human health has gradually attracted more and more attention, thus, calling for a systemic approach to evaluate the safety of metal-based NPs. Numerous data from *in vitro* and *in vivo* studies, epidemiological studies, and occupational health studies have shown the toxicity of NPs. Here, we summarized some standard tools used for nanotoxicity assessment (shown in Table 1). Owing to its low cost and simplicity, *in vitro* cellular experiments are routinely used to assess metel-based NP toxicity, allowing deeper investigation into molecular mechanisms. However, studies *in vitro* have apparent flaws, such as the inability to describe the distribution patterns of NPs *in vivo*. Thus its prediction power is limited. In contrast, *in vivo* animal studies have the advantage of estimating the toxic effects of metal-based NPs in complex physiological environments.

**TABLE 1:** Summary of some nanotoxicity assessment tools.

Levels	Toxicity tests	Methods
**In vitro**		
Cell culture	Cell viability	MTT (Lanone et al., 2009), Trypan blue assay (Cho et al., 2013)
Co-cultured cell lines	Oxidative stress	Fluorescence lifetime imaging microscopy (FLIM) (Balke et al., 2018) DCFH assay (Kroll et al., 2012; Wang et al., 2014; Wilson et al., 2015)
3D cell cultures	Apoptosis	Annexin V-PI assay (Wang et al., 2014)
	Inflammation	ELISA kit (detection of pro-inflammatory cytokines) (Kroll et al., 2012)
	Cell membrane integrity	LDH (lactate dehydrogenase) assay (Cho et al., 2013)
	Genotoxicity	Comet assay, micronucleus scoring with flow cytometry (Di Bucchianico et al., 2017)
	Mitochondrial Dysfunction	Mitochondrial Membrane Potential (MMP) detection using Tetramethylrhodamine (TMRM) (Wilson et al., 2015) or JC-1 (Wang et al., 2014) Mitochondrial morphology visualization by MitoTracker™ Red CMXRos (Wilson et al., 2015)
	Hemolytic Properties	Hemolysis assay (Dobrovolskaia et al., 2008)
**In vivo**		
Animal model	Pharmacokinetics and Biodistribution	Radiographic analysis, ICP-MS, transmission electron microscopy (TEM) (Almeida et al., 2011; He and Yoshioka, 2011)
	General condition	Weight loss, fatigue, loss of appetite, and change in fur color (Chen et al., 2009)
	Mortality	LD50 (Yu et al., 2013)
	Organ toxicity	Histopathology, ELISA, microscopy (Chen et al., 2009)
Human-level	—	Epidemiological investigations (Schulte et al., 2009)
	—	Occupational health assessment (Forest et al., 2021)
	—	Clinical trials for nanomedicine (Anselmo and Mitragotri, 2019)
**Omics**		
	Biomarker screening	Genomics, transcriptomics, proteomics, metabolomics, etc. (Frohlich, 2017)
**Computational level**		
Computer simulation	Toxicity prediction	Nano-QSAR (Puzyn et al., 2009); Data mining (Labouta et al., 2019) and Machine learning (Furxhi et al., 2019)

Since conventional toxicology studies are primarily based on one or two endpoints, this is insufficient to fully understand the mechanisms of metal-based NP toxicity. Recently, various high-throughput-based omics studies have been used to screen for NP toxicity, including genomics, transcriptomics, proteomics, metabolomics, etc., (Matysiak et al., 2016; Frohlich, 2017; Li et al., 2021). Omics technologies provide more comprehensive molecular profiling, and can be employed to screen NP toxicity at low exposure levels, and also have the advantage of identifying new biomarkers and targets of nanotoxicity both *in vivo* and *in vitro*. Omics approach also allows detection in miniscule variations within a cell compared to classical methods. However, the information obtained by a single omics approach is often far from complete, providing limited insight into complex molecular pathways and biological events in cells and organisms (Van Assche et al., 2015). Thus, the integration of omics has attracted more and more attention. And integrated omics was proven to help determine nanotoxicity. The combination of gene expression and metabolic profiling will provide more detailed and sensitive toxicological assessment of NPs (Shim et al., 2012). Metabolomics and transcriptomics combination facilitates more sensitive and detailed nano-toxicological assessments (Shin et al., 2018). Ruan et al. (2021) treated normal rat kidney cells with silica NPs (SiNPs) conducted a transcriptomic, proteomic and phosphoproteomic profiling, and established a new computational prediction algorithm of master autophagy-regulating kinases (cMAK) for integrating and analyzing multi-omics data. In this cMAK algorithm, the transcriptional, proteomic, and phosphoproteomic data of predicted kinases were wholly considered to reduce potentially false-positive hits, and predicted kinases with differentially regulated mRNAs at ≥2 time points, changed proteins at ≥1 time point, or altered phosphorylation sites at ≥1 time point were retained. Finally, 21 protein kinases candidates were predicted to be involved in autophage activation regulation induced by SiNPs. Since cMAK powerfully narrowed down the candidates, and then with the help of some additional experiments, two kinases, cyclin dependent kinase 4/7, were detected to be essential in SiNPs triggered autophage activation. Overall, omics analysis provides a promising future for systematically studying metabolic and physiological responses to NP exposure, with relatively low cost and high-dimensional data, and the integration of multi-omics can substantially improve the accuracy of the analysis.

Furthermore, there comes up a new way of predicting metal-based NP toxicity by computer simulation. This computational prediction relies on analyzing reliable experimental data of NPs structure, physicochemical properties and biological activity, as well as the measured toxicity (Richarz et al., 2017). Since computational chemistry can save time, funds and animal sacrifice compared to the traditional experiments, the quantitative structure–activity relationship (QSAR) approach may also be used for NP toxicity prediction. The idea of QSAR modeling of nanomaterials (Nano-QSAR) was introduced in 2009 by Puzyn et al. (2009). As Nano-QSAR model quantitatively describes the relationship in the form of mathematical equations, it is very dependent on the accuracy of the descriptors. The structure of metal-based NPs is highly complex and diversified, making it impossible to calculate theoretical descriptors (Li et al., 2022). Therefore, there comes up an urgent need to develop specific nano-descriptors to match the properties of metal-based NPs. And majority of the nano-QSAR models are based on the intrinsic physicochemical properties but ignoring the environmental influence (Puzyn et al., 2011; Kar et al., 2014), thus, it might be difficult to predict the toxicity directly from the structure. So, Anna *et al.* proposed to replace the traditional nano-QSAR model with an approach called Structure–Activity Prediction Networks (SAPNets), claiming it can effectively link NP structure with their toxicity through a series of layers built from nodes that correspond to predictive “meta-models” developed with machine learning techniques as well as Artificial Intelligence (Rybinska-Fryca et al., 2020). In contrast, the traditional nano-QSAR models mainly contains only one layer of descriptors, and it is not certain which descriptors are fundamental, and their application in predicting toxicity requires knowledge and experience in computational chemistry. However, the SAPNets are based on understandable descriptors (e.g., size, shape, aspect-ratio, and type of coating) and do not require additional computational calculations, allows consideration in environmental influence (such as solvent and pH), and the users can easily see how the change of the descriptor values will influence the predicted toxicity. Since computer simulations are highly dependent on previous experimental data, and the pathways and mechanisms of NPs toxic effects have not been fully elucidated, computer models may not be able to predict the toxic effects of NPs accurately. However, Gajewicz et al. (2017) proposed a quantitative read-across algorithm based on: one-point-slope, two-point formula, or the equation of a plane passing through three points, providing reliable predictions when only limited data is available. Labouta et al. (2019) developed a data mining method to assemble published evidence on NP cytotoxicity, with decision trees and feature selection algorithms, offering a powerful and relatively accurate tool for predicting cytotoxicity of NPs. Recently, machine learning techniques have also been applied in nanotoxicology. Furxhi et al. (2019) proposed a Copeland Index for machine learning to achieve more accurate predictions of NP toxicity for a given dataset. As more and more systematic data on NPs are available, data mining and machine learning are becoming powerful auxiliary tools for studying the toxic effects of NPs.

## Strategies to mitigate metal-based nanoparticle toxicity

**FIGURE 3:**
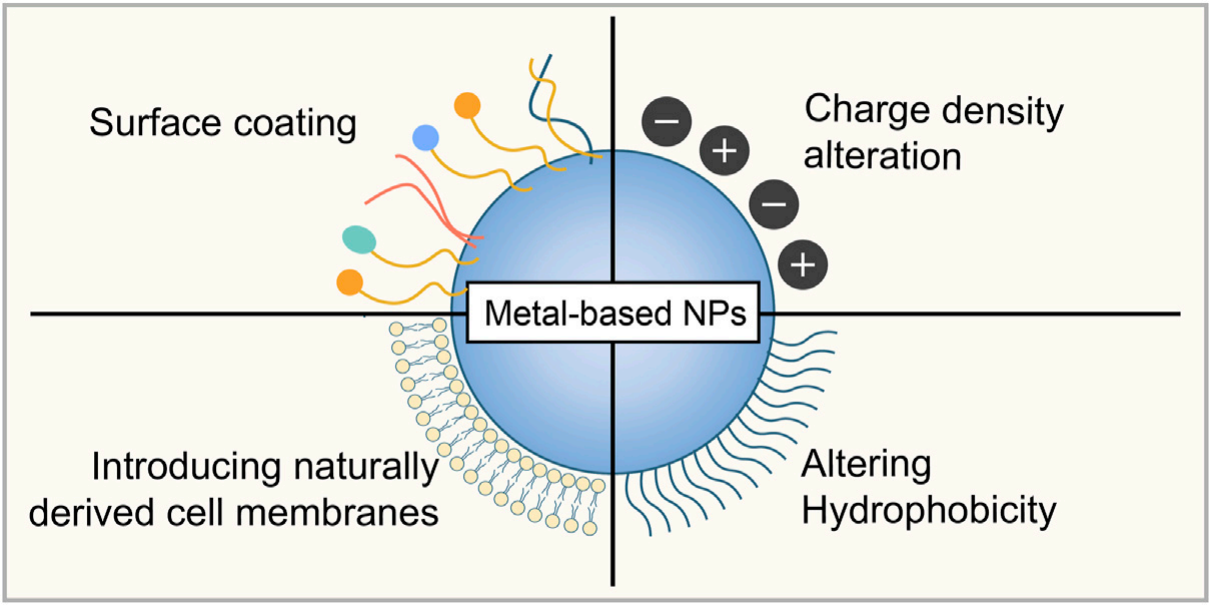
Strategies to mitigate metal-based NP toxicity. Surface coating, surface chemistry modifications by altering charge density and hydrophobicity, and introducing naturally derived cell membranes from several cell types can be used to reduce the potential toxicity of metal-based NPs.

Due to their unique properties, metal-based NPs have shown great potential in biomedical applications. Notably, NP toxicity remains a major obstacle for future applications. By understanding the mechanisms of metal-based NP toxicity, researchers have developed several strategies to mitigate their potential adverse impacts and toxicity, especially to achieve the goal of clinical translation of metal-based NPs. As the surface characteristics are primarily responsible for the toxicity of the NPs, modification of surface chemistry and properties has been reported to be one of the most common devised strategies to mitigate NP toxicity (Figure 3) (Amin et al., 2015).

Surface coating is one of the most popular surface modification strategies to reduce the potential toxicity of NPs, which is reversible by non-covalent modification (Hwang et al., 2018). Surface coating can alter the dispersion state of NPs, which significantly determines their bioavailability and potential toxicological effects (Wang et al., 2011). There are various types of coating materials such as polyethylene glycol (PEG), polyvinyl pyrrolidone (PVP), zwitterionic polymers, and poly (N -isopropyl acrylamide) (PNIPAM), and Polyvinyl alcohol (PVA) (Zhernenkov et al., 2015; Suk et al., 2016; Debayle et al., 2019; Lou et al., 2019; Ronavari et al., 2021). PEG is currently the most popular and practical material to passivate NP surfaces by shielding surface charges, imparting longer circulation time, and more cellular uptake of PEGylated NPs (Joralemon et al., 2010). Moreover, PEGylation of NPs can enhance their biocompatibility and reduce enzymatic degradation and non-immunogenicity (Karakoti et al., 2011). *In vitro* and *in vivo* experiments demonstrated no obvious toxicity of magnetic PEGylated Pt_3_Co NPs over a period of 60 days (Yin et al., 2013). Compared to conventional γ-Fe_2_O_3_ particles, no cytotoxicity and immunotoxicity were observed for PA-PEG@Fe_3_O_4_ and HA-PEG@Fe_3_O_4_ NPs in peripheral blood mononuclear cells (Patsula et al., 2019). A recent comparative study indicated that PEG-coated Au NPs induced less hepatotoxic outcomes than uncoated Au NPs in Sprague Dawley rats, which showed that PEG-coated Au NPs might be safer for biomedical applications (Patlolla et al., 2019).

Surface chemistry modifications by altering charge density and hydrophobicity have been reported to ameliorate NP toxicity and enhance the efficacy of NPs in biomedical applications (Mout et al., 2012; Nicol et al., 2015; Abd Ellah and Abouelmagd, 2017). The surface chemistry properties of NPs can be modified by covalently coupling with functional groups like anionic, nonionic, zwitterionic, and cationic groups onto the surface (Blanco et al., 2015; Zakharova et al., 2019; Han et al., 2020). These functional groups can influence the charge density and hydrophobicity of NPs (Nel et al., 2009; Aimonen et al., 2022). Au NPs can be an appropriate example where surface chemistry modification could influence NP toxicity. Au NPs functionalized with cationic groups were more toxic than NPs functionalized with anionic groups (Goodman et al., 2004). Furthermore, Fe_2_O_3_ NPs can generate ROS from the Fenton reaction on their surface, leading to cytotoxic effects (Voinov et al., 2011; Patil et al., 2015). The surface of Fe_2_O_3_ NPs can be functionalized with organic or inorganic materials to help stabilize iron oxidation, thereby reducing toxicity and improving biocompatibility (Wu et al., 2008; Zhu et al., 2018; Liu et al., 2020b). According to a recent study, ZnO NPs with hydrophoboic coatings had less toxic effects than those with hydrophilic coatings, possibly owing to the fact that hydrophobic surface coating could decrease bioavailability and thus reduce NP toxicity (Shukla et al., 2015; Lai et al., 2021).

There is a novel approach to introducing naturally derived cell membranes from several cell types such as red blood cells (RBCs), platelets, white blood cells (WBCs), and cancer cells onto the surface of NPs, enabling new properties like immune evasion, long-term circulation, specific recognition and targeting (Fang et al., 2018; Liu et al., 2021). Cell membranes are composed of lipids, proteins, and carbohydrates, which all play crucial roles in cellular signaling (Sarkar and Chattopadhyay, 2021). Further, cell membrane coatings can be employed to make NPs mimic the properties exhibited by the source cells, thereby directly replicating a variety of complex functions (Hu et al., 2015; Xia et al., 2019). The cell membrane coating technology was firstly reported using erythrocyte membranes for NP coating (Hu et al., 2011b). It was demonstrated that RBC membrane-coated NPs could effectively inhibit protein corona formation, which is known to promote biocompatibility, leading to their non-toxic or less toxic effects *in vivo* (Hu et al., 2011a; Rao et al., 2017). RBC membrane-coated Fe_3_O_4_ did not show significant *in vivo* toxicity in animal models by various assays, including blood biochemistry, whole blood panel examination, and histology analysis (Rao et al., 2016). This cell membrane coating strategy can effectively mitigate the potential toxicity of NPs for further safer biomedical applications.

## Current status of regulatory movement on nanotoxicity

Nanoscience has developed rapidly in the past 20 years, and many kinds of nanomaterials have been applied into clinical medicine. This raises to great concerns about the safety of nanomaterials, and puts forward higher requirements for the regulation of nanotoxicity. One of the main barriers to advancing nanotoxicology is the lack of harmonized and standardized NP characterization and risk assessment methods (Yang et al., 2021). The definition of NPs is still not universally acceptable. NPs are generally considered to be any particle with a size below 100 nm. But particles between 100–1,000 nm in size are also potentially toxic. Furthermore, the experimental design and data reporting are required to standardize to train predictive computational models. And the dose selected for NP toxicity assessment is also critical, especially in data mining and machine learning. In addition, the toxicity due to long-term low-dose NP exposure is often neglected, owing to the difficulties in simulating by traditional experimental models. The Nanotechnology Task Force (NTF) was launched in 2006 by FDA to help evaluate FDA’s regulatory authorities concerning the current state of nanotechnology. FDA had released a report about nanotechnology in 2020, showing the progress and innovation over a decade. And to date, FDA has issued six guidance documents regarding nanotechnology for industry, including one about drug products containing nanomaterials released in April 2022. FDA does not make definitive judgments about the nature of nanotechnology as safe or harmful. The regulatory approach of FDA is adaptive and flexible, considering the specific characteristics and effects of nanomaterials in the particular biological context of their intended use, as well as focusing on the interactions of nanomaterials with biological systems. To regulate and guide the research and evaluation of nanomedicine, the Center for Drug Evaluation (CDE) of the China National Medical Products Administration formulated the Technical Guidelines for Nanomedicines Non-Clinical Safety Evaluation Research (Trial), which was issued in August 2021 (Center for Drug Evaluation of Chinese National Medical Products Administration, 2021). This CDE guidance emphasized an appropriate selection of the test system, whether it is *in vitro* or *in vivo* or an alternative toxicity testing method, and also reminded to carefully choose the dose and testing time period. Furthermore, this guidance focused on the toxicity testing methods of nanomedicines on multiple systems including immunogenicity and immunotoxicity, neurotoxicity, genotoxicity, carcinogenicity, and reproductive toxicity. Although great efforts have been made by different regulatory agencies around the world like the United States FDA and China CDE to develop a proper guidance for the safer use of NPs, currently there are still no worldwide uniform and strict guidelines regarding NP toxicity testing (Saifi et al., 2018). Undoubtedly, the advances in the area of nanotoxicity study will ultimately facilitate to establish and implement a standardized and effective regulatory regime that harnesses the potential of nanotechnology and minimizes harm to humans.

## Conclusion

Since metal-based NPs are widely applicated in our daily life due to their unique properties, great attention has been attracted to their toxicity. Studies have shown that the toxic effects of NPs are mainly determined by several factors, such as physicochemical properties, dose, exposure pathways, and duration. Based on our current knowledge, it is challenging to elucidate the exact mechanism of metal-based NP toxicity. Recent studies have focused on oxidative stress as the underlying cause of metal-based NP toxicity. Indeed, as current *in vitro* and *in vivo* toxicity testing methods are mainly used to evaluate the acute and subacute toxicity of metal-based NPs, nanotoxicity testing methods for chronic long-term NPs exposure, which are crucial for predicting chronic toxicity in humans, are still lacking. Thus, there is an urgent need to develop new powerful tools to evaluate and understand the mechanisms of metal-based NP toxicity. Many promising effective strategies have been developed to design safer metal-based NPs for further biomedical applications to minimize NP toxicity. Ultimately, we should have a comprehensive understanding of metal-based NP toxicity before a general consensus can be reached on the toxicity of NPs. Shortly, we believe that the convergence of related disciplines like material science, medicine, chemistry, and artificial intelligence will significantly advance the development of nanotoxicity research, thereby making the application of metal-based NPs safer in humans.
